# Novel anti-infective potential of salvianolic acid B against human serious pathogen *Neisseria meningitidis*

**DOI:** 10.1186/s13104-016-1838-4

**Published:** 2016-01-13

**Authors:** Sanna Huttunen, Marko Toivanen, Chenghai Liu, Carina Tikkanen-Kaukanen

**Affiliations:** School of Pharmacy, University of Eastern Finland, Kuopio Campus, Kuopio, Finland; Institute of Public Health and Clinical Nutrition, University of Eastern Finland, Kuopio Campus, Kuopio, Finland; Shanghai University of Traditional Chinese Medicine, Shanghai, China; Ruralia Institute, University of Helsinki, Lönnrotinkatu 7, 50170 Mikkeli, Finland

**Keywords:** Anti-adhesion therapy, Antimicrobial, *Neisseria meningitidis*, Salvianolic acid B, Polyphenol

## Abstract

**Background:**

Epidemics of meningococcal meningitis cause significant health problems especially in Sub-Saharan Africa. Novel anti-infective candidates are needed. In modern anti-adhesion therapy initial attachment of bacteria to host cells is prevented. Our unique studies have revealed anti-adhesive candidates from natural products, namely milk and berries, against *Neisseria meningitidis* adhesion. In the present study against *N. meningitidis* adhesion, a novel binding inhibitor was found; salvianolic acid B (SA-B), a polyphenol from the radix *Salviae miltiorrhizae,* an important part of Chinese folk medicine.

**Methods:**

In order to test inhibition of meningococcal pili binding and anti-adhesion activity of SA-B, bovine thyroglobulin, a reference glycoprotein for meningococcal receptor was used in a microtiter plate assay. Inhibitory activity was tested by using serial dilutions of SA-B extracts of 98 and 70 % purity. Results were confirmed in a HEC-1B cell dot assay and antimicrobial activity was measured by using a microbroth dilution assay.

**Results:**

Almost total (93 %) inhibition of pili binding, anti-adhesion, was achieved with the 70 % extract of SA-B at the concentration of 0.3 mg/mL in the bovine thyroglobulin reference model. 50 % binding inhibition activity was achieved with 0.6 µg/mL of the SA-B extract. Total inhibition of the pili binding to HEC-1B cells was found at the tested concentration of 0.5 mg/mL. The 98 % pure SA-B resulted in weaker inhibition. At the concentration of 0.3 mg/mL 78 % inhibition was achieved in the thyroglobulin model. For 50 % inhibition 2.4 μg/mL of pure SA-B was needed. The difference between the binding inhibition activities (70 and 98 % pure SA-B) was statistically significant (P = 0.03). Antimicrobial activity of 70 % SA-B, when investigated against *N. meningitidis,* was detected only in relatively high concentrations.

**Conclusions:**

Our results indicate that plant SA-B may prevent meningococcal infections by inhibiting meningococcal binding and may thus have an impact on the amount of nasopharyngeal carriers of *N. meningitidis.* This may prevent the spreading of meningococcal infections between humans. One could conclude that SA-B and its source dried radix *S. miltiorrhizae,* which is an important part of Chinese folk medicine, could be valuable candidates for further research in meningococcal disease prevention.

## Background

Due to increasing bacterial resistance it is important to search for new means against bacterial infections [1]. In anti-adhesion therapy the adhesion of bacteria to host tissues is inhibited by soluble carbohydrates or their analogs [1–3]. Based on animal experiments soluble carbohydrates can protect against experimental infections [4]. Studies with natural products: milk, berries, berry polyphenols and herbal medicines have indicated these products being possible sources for anti-adhesive agents [5–14].

*Neisseria meningitidis,* which is an important human pathogen, colonizing the nasopharynx of 10–35 % of young adults, can be transmitted from person to person by droplet infection [15]. For a small amount of the colonised people meningococci can cause life-threatening infections, such as meningococcal septicaemia or meningitis [16]. Epidemics of meningitis are a significant health problem especially in Sub-Saharan Africa [17] but also amongst risk groups such as in military forces. The attachment of *N. meningitidis* to human mucosal epithelial cells, the crucial step of the infection [18], is mediated by type IV pili [19]. HEC-1B epithelial cell line [9, 19] and bovine thyroglobulin [5] have been previously used for adhesion and binding, as well as functioned as binding inhibition models for meningococcal pili.

Our previous studies have shown that in the thyroglobulin model oligosaccharides isolated from human and bovine milk have anti-adhesion activity against *N. meningitidis* [5]. We have also shown that in microtiter well binding and cell culture inhibition assays polyphenolic fractions extracted from berries possess anti-adhesive activity against *N. meningitidis* [6, 9].

**Fig. 1:**
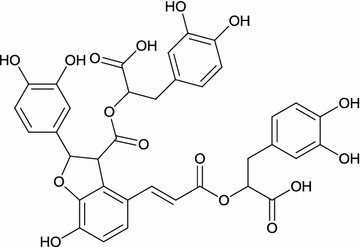
Molecular structure of SA-B

In the present study we tested anti-adhesion and antimicrobial activities of salvianolic acid B (SA-B) (Fig. 1), a water soluble polyphenolic acid extracted from dried radix *Salvia miltiorrhiza* Bunge, which is an important part of Chinese folk medicine. Over 20 *Salvia* species have been used in traditional Chinese folk medicine for the treatment of coronary heart diseases and strokes. According to Li et al. [20] only the official species of *S. miltiorrhiza* Bunge meet the requirements set forth and ascribed as the formal traditional medicinal plant Danshen.

SA-B has therapeutic potential against different medical conditions [21–23]. SA-B has been used in a clinical trial and has not displayed any obvious side effects [24]. To our knowledge this is the first report on anti-infective activity of SA-B and the first report on anti-adhesion activity of Chinese herbs against *N. meningitis.*

## Methods

### Materials

Biomax Ultrafree-15 centrifugal filter devises with 100 kDa cut offs were obtained from Millipore. d-biotinoyl-ε-aminocaproic acid-N-hydroxysuccinimide ester, streptavidin-POD conjugate, and ABTS-substrate were purchased from Roche diagnostics. Bovine thyroglobulin was obtained from Sigma. Polyvinylchloride microtiter plates (Falcon Flexible Plate) were from Becton–Dickinson Labware. Bacterial culture plates were purchased from Sarstedt and GCB agar from Oxoid Ltd. High-glucose DMEM cell culture medium, fetal bovine serum (FBS), l-glutamine, trypsin–EDTA solution and sterile phosphate buffered saline (PBS) for cell culture were from Gibco and cell culture plates from Greiner. Nitrocellulose membranes (Protran^®^) were obtained from Schleicher & Schuell BioScience. Super Signal solution was from Pierce and X-ray Hyperfilm™ from Amersham Pharmacia Biotech.

### Salvianolic acid B samples

Purified radix extract SA-B (98 % purity) was purchased from Ivy Fine Chemicals. SA-B extract of 70 % purity was purified from the radix *S. miltiorrhizae* by using ethanol extraction followed by column chromatography according to the method of Fung et al., 1993 [25]. Shortly: 500 g of *S. miltiorrhiza* Bunge was heated with 50 % ethanol (3 L) for 4 h. The mixture was allowed to cool down, after which it was filtered. After evaporation of ethanol the resulting aqueous solution was concentrated and allowed to stand overnight. After filtration, the aqueous solution was freeze-dried to a powder-like product (35 g). Column chromatography was then carried out by using C_18_ reverse phase column (Alltech, USA) with solvent gradient water to 80 % aqueous methanol. The separation was monitored by thin layer chromatography on silica gel (Merck, Germany) with solvent system n-butanol/acetic acid/H_2_0 and the purity was determined by using HPLC chromatography on C_18_ reverse phase column (Ultrasphere-ODS from Beckman, USA with 20 % aqueous CH_3_CN, UV 254 detector).

Initially both SA-B samples were tested for the binding inhibition activity against *N. meningitidis.* All the other experiments were carried out by using the SA-B of 70 % purity.

### Bacterial strain and culture conditions

*Neisseria meningitidis* serogroup C class I strain 8013 (X. Nassif, INSERM U570, Paris, France) was cultured at 37 °C in CO_2_ atmosphere for 18 h on GCB agar containing supplements described by Kellogg et al. [26].

### Isolation and biotin labelling of meningococcal pili

Isolation and biotin labelling of pili were carried out as described before [5, 6]. *N. meningitidis* bacteria were harvested from five plates and suspended into 20 mL of ice cold and sterile Hepes buffer (10 mmol/L). The suspension was mixed vigorously for 30 s and centrifuged (20 min at 4 °C, 8000*g*). The supernatant was applied to a Biomax Ultrafree-15 centrifugal filter device (cut off 100 kDa) and centrifuged (at 4 °C, 1000*g*). The concentrate was washed twice with 15 mL of Hepes buffer (10 mmol/L) and centrifuged as described above, to the volume of 1 mL. The concentrate was collected and stored at 4 °C. Biotin labelling of the isolated pili was performed in PBS by using d-biotinoyl-ε-aminocaproic acid-N-hydroxysuccinimide ester according to the instructions of the manufacturer. The biotin-labelled pili were stored at 4 °C.

### Inhibition of pili binding to bovine thyroglobulin

The inhibition of meningococcal pili binding to bovine thyroglobulin was tested as described before by using dry milk powder as a background control [6]. Aliquots of 100 µL of thyroglobulin (0.1 mg/mL) were incubated on polyvinylchloride microtiter plates at 4 °C overnight. The wells were washed five times with washing buffer [0.05 % (v/v) Tween 20 in PBS, pH 7.4]. The non-specific binding sites were blocked by dry milk powder solution [5 % (w/v) dry milk powder, 0.05 % (v/v) Tween 20 in PBS, pH 7.4], 250 µL/well. The plates were incubated for 1 h at room temperature. Biotin labelled pili were diluted 1:4 in PBS. Both of the SA-B samples were diluted with water to the concentration of 0.625 mg/mL, serially diluted down to the concentration of 0.00125 mg/mL and dilutions were separately mixed 1:1 with biotin-labelled pili. Pili-sample mixtures were incubated at room temperature for 1 h on a gentle rocking platform. Biotin labelled pili diluted 1:8 in PBS without herbal extract addition was treated identically to samples and used as a control. Then, 100 µL of the pre incubated solutions were added to washed, thyroglobulin-coated microtiter plate wells. After 1 h incubation at room temperature the wells were washed five times as described above, followed by Streptavidin-POD addition [100 µL of Streptavidin-POD conjugate in dry milk powder solution (1:4000)] and by 1 h incubation at room temperature. After five washes with washing buffer, an aliquot of 100 µL of ABTS-substrate was added. The absorbance was measured at 405 nm. The assays were always carried out in triplicates.

### Dot binding inhibition assay

Human epithelial HEC-1B cell line, a cell culture model for *N. meningitidis* [9, 19] was obtained from Xavier Nassif (INSERM U570, Paris, France). Cells were cultured on cell culture dishes in high-glucose DMEM supplemented with 10 % heat-inactivated FBS and 4 mM l-glutamine at 37 °C with 5 % CO_2_, without an addition of antibiotics. Cells from passages between 11 and 14 were harvested using a diluted trypsin–EDTA solution and centrifuged at 400*g*, 4 min. Cells were washed twice with ice cold sterile PBS, centrifuged as described above and stored at −20 °C until used.

In dot binding inhibition assay the anti-adhesion activity of SA-B was tested against meningococcal pili binding to HEC-1B cells. HEC-1B cells were collected from one plate as described above. They were suspended in 200 µL of PBS and diluted 1:10 with PBS. Cell suspensions of 2 µL were pipetted as dots on a nitrocellulose membrane and allowed to dry. Dry milk powder solution [5 % (w/v) dry milk powder, 0.05 % (v/v) Tween 20 in PBS, pH 7.4] was used to block the non-specific binding sites. After two hours of incubation (at room temperature) with gentle rocking on the rocking platform, the membrane was washed three times with washing buffer [0.05 % (v/v) Tween 20 in PBS, pH 7.4]. The 70 % SA-B sample was diluted with water to the concentration of 1 mg/mL. The diluted sample and biotin labelled diluted pili (1:10 in dry milk powder solution) were mixed 1:1 and preincubated for 1 h at room temperature. Labelled pili—sample mixture was added into the washed membrane and the incubation was performed for 90 min at room temperature. Biotin labelled pili dilution without herbal extract addition was treated identically to the sample and used as a positive control. After three washes with washing buffer, Streptavidin-POD conjugate diluted in dry milk powder solution (1:4000) was added into the membrane. The membrane was incubated for 1 h at room temperature. Finally, the membrane was washed and Super Signal Solution was added. The membrane was exposed to Hyperfilm™ and developed.

### Antimicrobial assay on microtiter plates

Antimicrobial activity of SA-B was tested against *N. meningitidis*. Overnight plate-cultured *N. meningitidis* bacteria were suspended in cold GC broth to an optical density of 0.180 at 550 nm. The corresponding colony forming unit (CFU) was 10^8^. Diluted bacterial suspension (90 µL) and SA-B dilutions ranging from 2 to 0.03 mg/mL (10 µL) were incubated on microtiter plate at 37 °C, in CO_2_ atmosphere, for 2 h. As a control, bacteria were incubated in the absence of SA-B or with ampicillin (0.1 mg/mL). The antimicrobial activity was analysed by plating the incubation mixtures in triplicate on GCB-agar plates. The survived colony forming units were counted next day. The growth inhibition was analysed by comparing the CFUs of bacteria–sample -mixture and control bacteria.

### Statistical analysis

Results for the inhibition assay were reported as mean ± standard deviation (SD). Two-tailed, unpaired Student’s t test (Microsoft Excel 2007, Microsoft Corp., Santa Rosa, CA, USA) was used for calculating the significance of the binding inhibition for both SA-B of 70 % purity and SA-B of 98 % purity compared to control, and for calculating the differences between inhibition caused by SA-B of 70 % purity and by SA-B of 98 % purity. The significance was defined as values of P < 0.05. Results for the antimicrobial assay were reported as mean ± standard deviation (SD). Two-tailed, unpaired Student’s t test (Microsoft Excel 2007, Microsoft Corp., Santa Rosa, CA, USA) was used for calculating the significance of the differences between CFUs from antimicrobial tests and control bacteria. Significance was defined as a value of P < 0.001.

## Results

### Anti-adhesive activity of the SA-B extracts

**Fig. 2:**
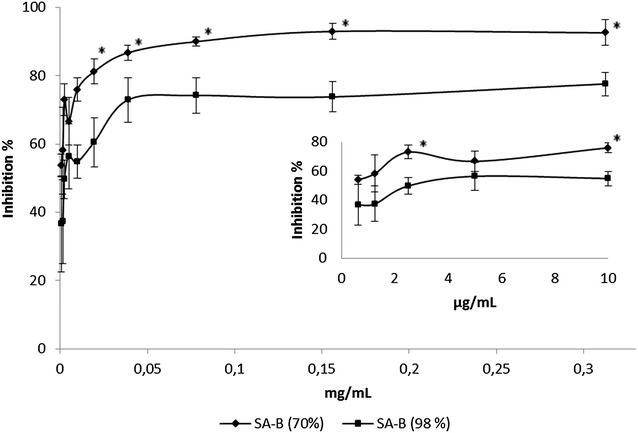
Inhibition of meningococcal pili binding to bovine thyroglobulin by salvianolic acid B (98 and 70 % purity). Tested concentrations ranged from 0.313 mg/mL to 0.6 µg/mL for both SA-Bs. Each point on the curves represents the mean ± SD, n = 9. The binding of pili to reference glycoprotein was significantly inhibited by both SA-B of 70 % purity (P < 0.001) and SA-B of 98 % purity (P < 0.001) when compared to control (no inhibitor). The difference between the inhibition activity caused by SA-B of 70 % purity compared to SA-B of 98 % purity was significant (*P = 0.03, except for the concentration of 0.002 mg/mL *P = 0.01)

**Fig. 3:**
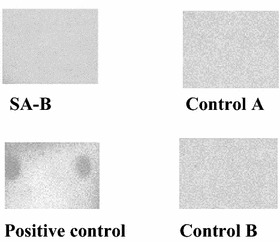
Inhibition of meningococcal pili binding to HEC-1B human epithelial cells by SA-B. Positive control shows pili biding without presence of SA-B. Controls A and B do not contain pili or Streptavidin-POD, respectively

To demonstrate the SA-B anti-adhesive activity, both SA-B of 98 % purity and SA-B of 70 % purity were tested. The binding inhibition for both SA-B of 70 % purity (P < 0.001) and SA-B of 98 % purity (P < 0.001) was significant compared to control. Binding inhibition activity of the SA-B with 98 % purity was 78 % at the concentration of 0.3 mg/mL, and concentration for the 50 % inhibition was 2.4 µg/mL. The SA-B extract of 70 % purity inhibited the pili binding almost totally (93 %) at the concentration of 0.3 mg/mL and 50 % inhibitory activity was achieved at the concentration of 0.6 µg/mL. Quite similar results in the binding inhibition activity were achieved with both extracts, however the SA-B of 70 % purity was significantly (P = 0.03) more efficient in binding inhibition than SA-B of 98 % purity (Fig. 2). In the lowest concentrations (below 0.01 mg/mL) the difference was non-existent, except at the concentration of 0.002 mg/mL (P = 0.01). When the 70 % SA-B (0.5 mg/mL) extract was tested for the inhibition of meningococcal pili binding to HEC-1B epithelial cells, the extract was able to completely inhibit the attachment of meningococcal pili (Fig. 3).

### Antimicrobial activity of the SA-B

**Fig. 4:**
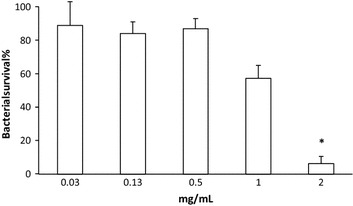
Antimicrobial activity of SA-B against *Neisseria meningitidis*. Bacterial survival compared with control, mean ± SD of three experiments. *P < 0.001 against the bacterial control

Antimicrobial activity has been found amongst berries, herbs and berry phenolics [27–29]. In antimicrobial assay the growth of *N. meningitidis* was inhibited effectively (P < 0.001) in relatively high concentrations (2 mg/mL) of the 70 % SA-B. Other tested SA-B concentrations (1–0.03 mg/mL) did not have significant effect on meningococcal growth and the bacterial survival was found to be between 58 and 89 %, respectively. Bacterial survival with ampicillin was 23 % (Fig. 4).

## Discussion

Even if the herbal solution is efficacious, immediate determination of the active substances may not be possible, particularly in herbal solutions that contains many compounds. Components in a crude extract could act synergistically, have unknown interactions and interact to diminish possible adverse effects of the components. Also, if used properly, a mixture of several crude extracts could have greater beneficial effects compared to a single plant extract. This has been shown by using Chinese herbs against hemagglutination and adhesion of *Escherchia coli* [30].

*Neisseria meningitidis* adhesion is mediating by type 4 pili, which carries two main potential adhesins [31, 32], but the adhesion mechanisms are complicated [33]. The SA-B sample of 70 % purity may contain components that interact with different binding activities of *N. meningitidis*. This may be one explanation for higher binding inhibitory activity of 70 % SA-B compared to 98 % SA-B. Our previous results suggest carbohydrate recognition for *N. meningitidis* and binding inhibition by milk oligosaccharides [5]. Parallel to results achieved here we have also shown that berry extracts rich in polyphenols are active against pili binding [6, 9].

Bacterial binding to host tissues is an important phase before bacterial infections [1, 2]. In this study we found binding inhibition activity of SA-B against meningococcal pili, which mediate *N. meningitidis* adhesion. There is a lack of proper vaccination against *N. meningitidis* [17]. Natural products as anti-adhesives could offer an important solution for prevention. As an anti-adhesive agent SA-B could possibly reduce the number of nasopharyngeal carries and thus reduce the risk of spreading the infection. The use of known, natural nutritional products in a form of an extract rather than a purified compound would be practical and economic for prevention purposes. This could be useful especially in developing countries, where proper preservation of vaccines is limited and epidemics of meningitis are a major health problem.

Here we show the inti-infective, anti-adhesive effect of SA-B. In anti-adhesion therapy the interaction between the pathogen and the host is inhibited without killing the bacteria thus avoiding rise of bacterial resistance [34]. Antimicrobial activity of the SA-B was only found in higher and not in lower concentrations, and in concentrations possessing binding inhibitory activity. The inhibitory activity found in the present study was not antimicrobial but anti-adhesive indicating SA-B as a candidate for anti-adhesion therapy against *N. meningitidis.*

## Conclusions

Our results indicate that both 70 and 98 % pure SA-B extracts may prevent meningococcal infections by inhibiting meningococcal binding to host cells. It can be assumed that this inhibition may also have a reducing impact on the amount of nasopharyngeal carriers of *N. meningitidis* and thus preventing the spread of meningococcal infections between humans. One could conclude that SA-B and its source, dried radix *Salviae miltiorrhizae,* an important part of Chinese folk medicine, could be valuable candidates for further research in meningococcal disease prevention.

## Abbreviations

CFU: colony forming unit
SA-B: salvianolic acid B
